# Aqueous humor proteome of primary open angle glaucoma: A combined dataset of mass spectrometry studies

**DOI:** 10.1016/j.dib.2020.106327

**Published:** 2020-09-21

**Authors:** W.H.G Hubens, R.J.C Mohren, I Liesenborghs, L.M.T Eijssen, W.D Ramdas, C.A.B Webers, T.G.M.F Gorgels

**Affiliations:** a University Eye Clinic Maastricht, Maastricht University Medical Center, Maastricht, the Netherlands; b Department of Mental Health and Neuroscience, Maastricht University, Maastricht, the Netherlands; c Maastricht MultiModal Molecular Imaging (M4I) Institute, Division of Imaging Mass Spectrometry, Maastricht University, Maastricht, the Netherlands; d Maastricht Centre of Systems Biology (MaCSBio), Maastricht University, Maastricht, The Netherlands; e Department of Bioinformatics - BiGCaT, NUTRIM, Maastricht University, Maastricht, the Netherlands; f Department of Ophthalmology, Erasmus Medical Center, Rotterdam, the Netherlands

**Keywords:** Primary open angle glaucoma, Aqueous humor, Human, Proteome, Liquid chromatography tandem mass spectrometry

## Abstract

Analysis of the proteins of the aqueous humor can help to elucidate the complex pathogenesis of primary open angle glaucoma. Thanks to advances in liquid chromatography tandem mass spectrometry (LC-MS/MS) it is now possible to identify hundreds of proteins in individual aqueous humor samples without the need to pool samples. We performed a systematic literature search to find publications that performed LC-MS/MS on aqueous humor samples of glaucoma patients and of non-glaucomatous controls. Of the seven publications that we found, we obtained the raw data of three publications. These three studies used glaucoma patients that were clinically similar (i.e. undergoing glaucoma filtration surgery) which prompted us to reanalyse and combine their data. Raw data of each study were analysed separately with the latest version of MaxQuant (version v1.6.11.0). Outcome files were exported to Microsoft Excel. Samples belonging to the same patient were averaged to obtain peptide expression values per individual. We compared the overlap of identified proteins using the VLOOKUP function of Excel and a publicly available Venn diagram software. For the peptide sequences that can belong to multiple proteins (usually of the same protein family), we initially included all possibly identified proteins. This ensured that we would not miss a potential overlap between the studies due to differences in identified peptide counts. Next, of those peptides of which we compared multiple proteins, only one unique protein was included in our analysis i.e. either the protein overlapping between studies or in case of no overlap, the protein that had the highest identified peptide count. This yielded 639 unique proteins detected in aqueous humor of either glaucoma patients or non-glaucomatous controls. In our manuscript entitled “The aqueous humor proteome of primary open angle glaucoma: An extensive review” [1], we further analysed this dataset. The dataset was exported to Perseus (version 1.6.5.0). We removed contaminants and filtered for proteins detected with high confidence, i.e. in more than 70% of the samples of at least one study. This yielded 248 proteins of which we compared the expression in glaucoma patients against control patients. Gene ontology enrichment analysis and pathway analysis was used to interpret the results. The unfiltered dataset reported in this data article and the approach reported here to reanalyse and combine raw data of different studies can be applied by other glaucoma researchers to gain more insight in the pathogenesis of glaucoma.

## Specifications Table

**Table:** 

Subject	Ophthalmology
Specific subject area	Aqueous humor proteome of primary open angle glaucoma
Type of data	Table
How data were acquired	Raw data were obtained from ProteomeXchange, a publicly available database and reanalysed with the freely available MaxQuant software (Max Planck Institute version v1.6.11.0). During our study, dataset PXD004928 was not yet publicly available and we obtained the raw data after contacting the authors. Microsoft Excel was used to combine the files. Subsequently, we imported the combined dataset in Perseus (Max Planck Institute version 1.6.5.0) for filtering and statistical analysis.
Data format	Raw Analysed Filtered
Parameters for data collection	We performed a systematic literature search to find studies that investigate the proteome of aqueous humor from patients with glaucoma compared to non-glaucomatous controls. We considered only studies that included glaucoma patients without other ocular comorbidities. This meant that from the 9 proteomic studies we found, 7 were eligible to obtain the raw data. We managed to obtain the raw data of three publications. They used similar glaucoma patients i.e. patients undergoing glaucoma filtration surgery, which prompted us to reanalyse their raw data and combine the outcome for new statistical analysis.
Description of data collection	We reanalysed the raw data of three publications that investigated the aqueous humor proteome of primary open angle glaucoma patients compared to non-glaucomatous controls, using LC-MS/MS. We downloaded the raw data from the depositories and subsequently loaded them into the MaxQuant software program (v1.6.11.0) for analysis. Analysed data were exported to Microsoft Excel to average duplicates and to combine the different studies into 1 protein database. This database was imported into Perseus analysis software (v1.6.5.0) to filter for proteins with high detection confidence and subsequent statistical analysis to compare glaucoma patients with controls.
Data source location	University Eye Clinic Maastricht Maastricht Netherlands
Data accessibility	RAW data were obtained from ProteomeXchange: Dataset 1: “Human aqueous humor of Primary open angle glaucoma LC-MS/MS”; PXD007624; https://www.ebi.ac.uk/pride/archive/projects/PXD007624 Dataset 2: “Comparative shotgun proteomics of aqueous humor for cataract, glaucoma and pseudoexfoliation eye disorders”; PXD002623; https://www.ebi.ac.uk/pride/archive/projects/PXD002623 Dataset 3: “Comparative evaluation of the aqueous humor proteome of primary angle closure and primary open angle glaucomas and senile cataract eyes”; PXD004928; https://www.ebi.ac.uk/pride/archive/projects/PXD004928 Analysed data are included in this article
Related research article	WHG Hubens, RJC Mohren, I Liesenborghs, LMT Eijssen, WD Ramdas, CAB Webers, TGMF Gorgels, The aqueous humor proteome of primary open angle glaucoma: an extensive review, Exp. Eye Res. 197 (2020) 108077 doi:10.1016/j.exer.2020.108077

## Value of the Data

• This dataset provides the list of proteins present in the aqueous humor of primary open angle glaucoma patients and cataract patients and facilitates extraction and quantification of disease specific differences.
• This dataset is a rich resource for glaucoma researchers and pharmaceutical companies interested in unravelling the proteome of primary open angle glaucoma.
• The dataset facilitates pathway analysis to identify new glaucoma pathways that can be targeted in human or animal studies, with the aim of establishing new biomarkers or new interventions for primary open angle glaucoma.
• The approach detailed here to regroup, combine and reanalyse publicly available data may be useful for other studies on data in public databases.

## 1 Data Description

Fig. 1:

**Fig. 1:**
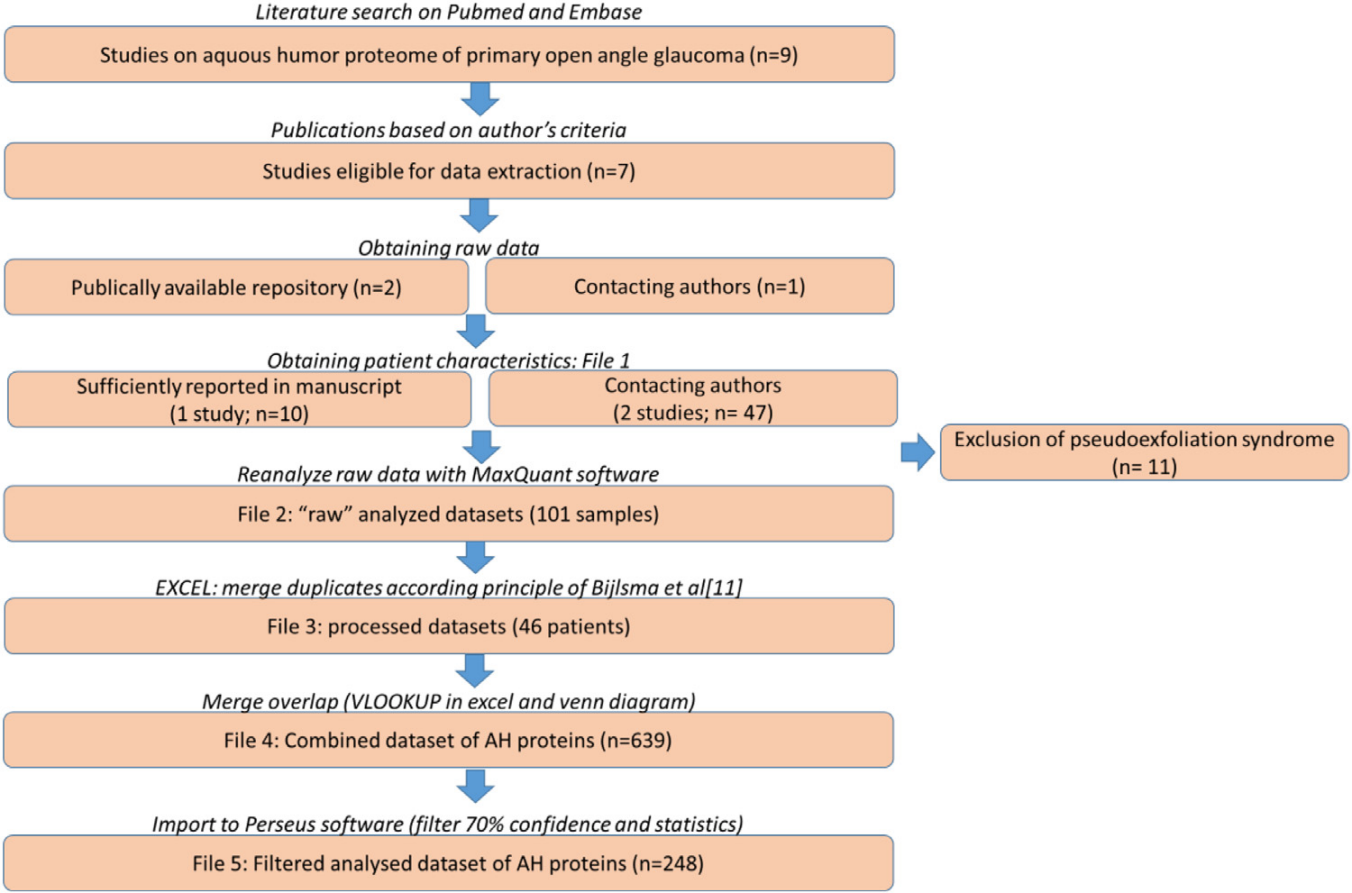
Flowchart reporting the workflow to obtain a combined proteomic dataset of glaucomatous aqueous humor.

Fig. 1 is a flowchart that visualizes the workflow that we followed in our review on the aqueous humor proteome of primary open angle glaucoma patients. In short, a literature search was performed to find eligible studies. We subsequently tried to obtain the raw data related to these studies either via publicly available repositories or by attempting to contact the corresponding author. Three datasets were obtained (see data accessibility table for the respective links). Each dataset was reanalysed and processed, after which they were combined into 1 dataset for statistical analysis.

File 1:

File 1 is a description of the patient characteristics. The columns are self-explanatory. Humphrey visual field analyser test results (column J and K) were not available for some patients as indicated by “NA”. The samples highlighted in red were excluded from our combined analysis, because these patients were additionally diagnosed with pseudoexfoliation syndrome (PEX). The remaining controls and glaucoma patients were pooled to form a combined dataset of which the average age, gender distribution and average eye pressure is provided in columns Q-S. Statistical analysis (column T) showed that these parameters were not significantly different between the two groups.

File 2 (general):

We reanalysed the three datasets with MaxQuant and exported the output files to Microsoft Excel (file 2). The data are named after the corresponding first authors. These files are considered as raw data, i.e. they are unprocessed and contain several redundant columns. The general layout is as follows: possible identified proteins (A), protein with most peptide reads (B), how many times a peptide was measured (C-E), the protein names (F), gene symbol (G), fasta header (H), peptide read per sample, molecular weight of the protein, peptide identification method, sequence coverage, uncorrected intensity, IBAQ correction intensity, LFQ corrected intensity and MS/MS count.

For each file, the samples were differently annotated by the authors. An overview is given below:

File 2 dataset 1: Adav.

This dataset contains 5 control patients (CG065, CG070, CG072, CG075 and CG078) and 5 glaucoma patients (G009, G010, G016, G039 and G041). Aqueous humor of each patient was analysed in duplo (_A and _B).

File 2 dataset 2: Kaur.

This dataset contains 9 control patients and 9 glaucoma patients. Control group was denoted as Cat and glaucoma group as POAG. It seems this study was performed in two batches. The first batch of 4 control and 4 POAG patients was annotated as “long” (Cat 1 U R long, Cat 2 long, Cat 3 long, Cat 4 long, POAG 1 long, POAG 2 long, POAG 3 long ad POAG 4 long) and was performed in duplo (long1 vs long2). One sample was also analysed a third time (cat 1 U R) presumable to test a different protocol. The second batch of 5 control (New Cat 1–5) and 5 POAG (New POAG 1–5) were not measured in duplo.

File 2 dataset 3: Kliuchnikova.

This dataset contains 11 control patients (k10, k14, k18, k24, k32, k44, k52, k60, k62, k64, k8) and 7 glaucoma patients (g110, g114, g116, g12, g50, g54, g56). All patients were analysed in triplo (_1, _2 and _3).

Processed datasets (file 3 and file 4):

Protein expressions of duplicate samples were averaged. The averaged intensity, iBAQ intensity and LFQ intensity for each dataset are provided in file 3. This file contains three tabs named “Adav_duplo removed”, “Kaur_duplo removed” and “Kliu_duplo removed”. Layout and sample coding is the same as for file 2. Using VLOOKUP function of Microsoft Excel and Venn diagram software all reported proteins across studies were matched into a single file (file 4). We present the proteins (A), majority protein UniProt ID (B), protein name (C), gene name (D), fasta header (E), in how many samples the protein is identified within each group and study (G-L), the average LFQ expression in each study (N-P) and showed that after normalization the average LFQ intensity was the same in each study (column S-U). The normalized LFQ intensity per sample/study is reported (column W-BP) and the raw LFQ intensities is presented in column BR-DK. Raw intensities (DR-FK) and iBAQ normalized intensities (FP-HI) are also provided.

Filtered dataset (file 4):

For the purpose of our review [1], the dataset was further analysed in Perseus. We removed contaminants, filtered on proteins whose LFQ protein expression was detected in more than 70% of the samples in at least one study, log-normalized the LFQ intensities and performed multiple ANOVA to compare glaucoma and control patients. The outcome was again exported to Microsoft Excel (file 4). The filtered data file consists of the following columns: gene name (A), majority protein Uniprot ID (B), protein name (C), mean expression in controls (D), in how many control samples the protein was detected (E), mean expression in glaucoma (F), in how many glaucoma samples the protein was detected (G), and difference in log transformed protein expression between glaucoma and controls (H). The uncorrected p-value (I) and the FDR-corrected q-value (J) are reported. Column L-BE are protein expression values of each individual sample.

## 2 Experimental Design, Materials, and Methods

As depicted in the flowchart (fig. e1), we performed a systematic literature search to find studies that reported proteomics data from LC-MS/MS studies of glaucoma aqueous humor samples. Keywords used were “primary open angle glaucoma” and “aqueous humor”. We found 9 LC-MS/MS studies of which 7 studies matched our criteria that other ocular diseases are absent [2], [3], [4], [5], [6], [7], [8]. We attempted to get access to the underlying raw data either via depositories or by contacting the corresponding authors. We managed to obtain the raw data of three publications [2], [3], [4] (PXD007624, PXD002623 and PXD004928).

Of two of these publications, the patient characteristics were unfortunately not well defined. Upon contacting the corresponding authors, they kindly provided us the missing information. We report the detailed patient characteristics in this manuscript (file 1). Since the inclusion and exclusion criteria were largely overlapping between the three studies, we decided to pool the controls and to pool the glaucoma patients for a combined analysis. Patients additionally diagnosed with pseudoexfoliation syndrome were excluded from this combined dataset. As seen from columns Q-T, the pooled group of 25 controls and 21 glaucoma patients were comparable in terms of age, gender distribution and eye pressure.

The raw data of primary open angle glaucoma patients and controls were reanalyzed using MAXQuant software (Max Planck Institute; [9,10]). As the raw data varied greatly between the studies, we failed to normalize the data in a pooled reanalysis. Therefore, we decided to reanalyze each study separately. The following settings were used:

• Variable modification: Oxidation (M) and Acetylation (protein N-term)
• Fixed modification: Carbamidomethyl (C)
• Trypsin digestion
  ○ Max missed cleavage: 2
• Label free quantification
  ○ Minimum ratio count: 2
  ○ Fast LFQ mode enabled,
  ○ Stabilize large LFQ ratios
  ○ Min number of neighbours: 3; average number of neighbours: 6
• Peptide identification:
  ○ “from and to”
  ○ Advanced identification enabled
    ■ Second peptides
    ■ Match between runs

Output files were exported to Microsoft Excel (file 2). Sample or run duplicates were combined to obtain protein expression values per individual (file 3). We did this according the data processing recommendations of Bijlsma et al [11]. This meant that samples were averaged if more than one sample had LFQ expression values. If only one of the duplicate samples had measured expression values, this sample was considered as the average. For proteins of which none of the replicates had expression values, the value was set to 0. To combine the datasets, we extracted the list of majority protein ID's from each study. In case of multiple majority protein IDs matching to a peptide sequence, we separated them into different columns. This enabled us to check if at least one of the suggested proteins was reported in the other studies, ensuring the highest amount of overlap between the studies. We identified the overlap via two different methods i.e. the VLOOKUP function of Microsoft Excel and by using a free Venn diagram software (VIB-Ugent; http://bioinformatics.psb.ugent.be/webtools/Venn/). After we established what proteins had overlapping detection between studies we used the VLOOKUP function to copy the corresponding expression values of each study, creating our final combined dataset (file 4). For combined analysis in the publication corresponding to this dataset [1], we used the LFQ intensities of the proteins. LFQ intensities varied greatly between studies (1000 fold difference) and needed normalization. This was achieved by dividing the LFQ intensity of a protein by the average LFQ intensity in the respective study and then multiplying by the average LFQ intensity across all studies. Researchers can apply other normalization methods on this dataset for intensity, iBAQ intensity and LFQ intensity. File 4 was subsequently imported to a free analysis software (Perseus 1.6.5.0; Max Planck Institute) [12]. Here we filtered for proteins that were not considered contaminants and were detected with a high confidence. This meant that within a study, proteins were detected in at least 70% of either the control patients or the glaucoma patients. Next, we performed a log-transformation on the normalized LFQ protein expression intensity data and statistically compared the expression of the glaucoma group and the control group using the build in multiple comparison ANOVA with FDR-adjusted correction. The outcome was exported back to Microsoft Excel (file 5).

## 3 Ethics Statement

The current study used data from three previously published datasets on human aqueous humor proteome and we did not have contact with any of the study participants. All studies declared that they adhered to the Declaration of Helsinki and performed the studies on participants that provided written informed consent.

## Declaration of Competing Interest

The authors declare that they have no known competing financial interests or personal relationships which have, or could be perceived to have, influenced the work reported in this article.

## Appendix Supplementary materials

Image, application 1

## References

[1] Hubens WHG, Mohren RJC, Liesenborghs I, Eijssen LMT, Ramdas WD, Webers CAB, Gorgels TGMF (2020). The aqueous humor proteome of primary open angle glaucoma: an extensive review. Exp. Eye Res..

[2] Adav SS, Wei J, Terence Y, Ang BC, Yip LW, Sze SK (2018). Proteomic analysis of aqueous humor from primary open angle glaucoma patients on drug treatment revealed altered complement activation cascade. J. Proteome. Res..

[3] Kaur I, Kaur J, Sooraj K, Goswami S, Saxena R, Chauhan VS, Sihota R (2018). Comparative evaluation of the aqueous humor proteome of primary angle closure and primary open angle glaucomas and age-related cataract eyes. Int. Ophthalmol..

[4] Kliuchnikova AA, Samokhina NI, Ilina IY, Karpov DS, Pyatnitskiy MA, Kuznetsova KG, Toropygin IY, Kochergin SA, Alekseev IB, Zgoda VG (2016). Human aqueous humor proteome in cataract, glaucoma, and pseudoexfoliation syndrome. Proteomics.

[5] Salamanca D, Gomez-Chaparro JL, Hidalgo A, Labella F (2018). Differential expression of proteome in aqueous humor in patients with and without glaucoma. Arch. Soc. Esp. Oftalmol..

[6] Ji Y, Rong X, Ye H, Zhang K, Lu Y (2015). Proteomic analysis of aqueous humor proteins associated with cataract development. Clin. Biochem..

[7] Kaeslin MA, Killer HE, Fuhrer CA, Zeleny N, Huber AR, Neutzner A (2016). Changes to the aqueous humor proteome during glaucoma. PLoS One.

[8] Sharma S, Bollinger KE, Kodeboyina SK, Zhi W, Patton J, Bai S, Edwards B, Ulrich L, Bogorad D, Sharma A (2018). Proteomic alterations in aqueous humor from patients with primary open angle glaucoma. Invest. Ophthalmol. Vis. Sci..

[9] Ox J, Mann M (2008). MaxQuant enables high peptide identification rates, individualized p.p.b.-range mass accuracies and proteome-wide protein quantification. Nat. Biotechnol..

[10] Tyanova S, Temu T, Cox J (2016). The MaxQuant computational platform for mass spectrometry-based shotgun proteomics. Nat. Protocols.

[11] Bijlsma S, Bobeldijk I, Verheij ER, Ramaker R, Kochhar S, Macdonald IA, van Ommen B, Smilde AK (2006). Large-scale human metabolomics studies: a strategy for data (pre-) processing and validation. Anal. Chem..

[12] Tyanova S, Temu T, Sinitcyn P, Carlson A, Hein MY, Geiger T, Mann M, Cox J (2016). The Perseus computational platform for comprehensive analysis of (prote)omics data. Nat. Methods.

